# **Inhibition of retinoid X receptor improved the morphology, localization of desmosomal proteins** **and paracellular permeability in three-dimensional cultures of mouse keratinocytes**

**DOI:** 10.1093/jmicro/dfac007

**Published:** 2022-02-16

**Authors:** Shoko Ishikawa, Misaki Nikaido, Takahito Otani, Kayoko Ogata, Hiroshi Iida, Yuko Inai, Sachio Tamaoki, Tetsuichiro Inai

**Affiliations:** Department of Oral Growth and Development, Fukuoka Dental College, 2-15-1 Tamura, Sawara-ku, Fukuoka 814-0193, Japan; Department of Odontology, Fukuoka Dental College, 2-15-1 Tamura, Sawara-ku, Fukuoka 814-0193, Japan; Department of Morphological Biology, Fukuoka Dental College, 2-15-1 Tamura, Sawara-ku, Fukuoka 814-0193, Japan; Department of Morphological Biology, Fukuoka Dental College, 2-15-1 Tamura, Sawara-ku, Fukuoka 814-0193, Japan; Oral Medicine Research Center, Fukuoka Dental College, Fukuoka 814-0193, Japan; Laboratory of Zoology, Graduate School of Agriculture, Kyushu University, 744 Motooka, Nishi-ku, Fukuoka 819-0395, Japan; Division of General Dentistry, Kyushu University Hospital, 3-1-1 Maidashi, Higashi-ku, Fukuoka 812-8582, Japan; Department of Oral Growth and Development, Fukuoka Dental College, 2-15-1 Tamura, Sawara-ku, Fukuoka 814-0193, Japan; Department of Morphological Biology, Fukuoka Dental College, 2-15-1 Tamura, Sawara-ku, Fukuoka 814-0193, Japan; Oral Medicine Research Center, Fukuoka Dental College, Fukuoka 814-0193, Japan

**Keywords:** retinoic acid, retinoid X receptor, keratinocyte, three-dimensional culture, desmosome, tight junction

## Abstract

Retinoic acid (RA) plays an important role in epithelial homeostasis and influences the morphology, proliferation, differentiation and permeability of epithelial cells. Mouse keratinocytes, K38, reconstituted non-keratinized stratified epithelium in three-dimensional (3D) cultures with serum, which contains retinol (a source of RA), but the morphology was different from *in vivo* epithelium. The formed epithelium was thick, with loosened cell–cell contacts. Here, we investigated whether the inhibition of RA receptor (RAR)/retinoid X receptor (RXR)-mediated signaling by an RXR antagonist, HX 531, improved K38 3D cultures in terms of morphology and intercellular junctions. The epithelium formed by 0.5 μM HX531 was thin, and the intercellular space was narrowed because of the restoration of the layer-specific distribution of desmoglein (DSG)-1, DSG3 and plakoglobin (PG). Moreover, the levels of desmosomal proteins and tight junction proteins, including DSG1, DSG2, DSG3, PG, claudin (CLDN)-1 and CLDN4 increased, but the adherens junction protein, E-cadherin, did not show any change. Furthermore, CLDN1 was recruited to occludin-positive cell–cell contacts in the superficial cells and transepithelial electrical resistance was increased. Therefore, K38 3D cultures treated with 0.5 μM HX531 provides a useful *in vitro* model to study intercellular junctions in the non-keratinized epithelium.

## Introduction

Retinol (vitamin A), an essential nutrient, plays a role in the regulation of epithelial cell homeostasis, including cell differentiation, proliferation, morphogenesis and maintenance of the epithelium [1]. Retinol in the serum is taken up by the cells and metabolized to retinoic acid (RA) via retinaldehyde [2]. All-trans-retinoic acid (atRA), an isomer of RA, including 9-cis-RA (9cRA) and 13-cis-RA (13cRA), is the major physiologically active retinol metabolite. Retinoic acid receptors (RAR) α, β, γ and retinoid X receptors (RXR) α, β, γ are nuclear receptors that bind to RA. While atRA binds only to RAR, 9cRA binds to both RAR and RXR [3,4]. Ligand-bound RAR/RXR heterodimers bind to retinoic acid response elements in the regulatory regions of target genes to alter gene transcription [5,6]. Moreover, RXR can form heterodimers with the peroxisome proliferator-activated receptor (PPAR-α, γ, δ), liver X receptor (LXR-α, β), farnesoid X receptor, pregnane X receptor, thyroid hormone receptor (TR-α, β) and vitamin D receptor (VDR). RXR can also form homodimers and homotetramers [7].

The epidermis (keratinized stratified squamous epithelium) and oral mucosal epithelium (non-keratinized stratified squamous epithelium) function as structural and functional barriers against dehydration, microorganisms, physical stimulations and chemical substances. Keratinocytes, the principal cells of the epidermis and oral mucosal epithelium, are connected to each other by intercellular junctions, such as adherens junctions (AJs), desmosomes (DSMs) and tight junctions (TJs), which form a physical barrier to close the intercellular space.

E-cadherin and desmogleins (DSGs)1–4 are transmembrane calcium-dependent cell adhesion proteins, which are localized in keratinocyte cell–cell contacts (AJs and DSMs, respectively) in the epidermis and oral mucosal epithelium, and belong to the cadherin superfamily. Plakoglobin (PG) interacts with both E-cadherin and DSGs and interacts with α-catenin and desmoplakin, which are actin and cytokeratin intermediate filament binding proteins, respectively [8]. E-cadherin is localized throughout the epithelium, except for the cornified layer, while DSG1 and DSG3 are distinctly localized in the epidermis and oral mucosal epithelium [9,10]. In the epidermis, DSG1 was localized throughout the epidermis, more intense in the superficial layers but less intense in the deep basal layer, while DSG3 was limited to the basal and most immediate suprabasal cells. In the oral mucosal epithelium, DSG1 was detected throughout the epithelium, much more intense in the superficial layers, whereas DSG3 was localized throughout the mucosal epithelium [10].

TJs form a paracellular permeability barrier that regulates the passage of solutes and water through the paracellular pathway. Claudin (CLDN), occludin (OCLN) and tricellulin are transmembrane proteins of TJs, and CLDNs, more than 20 members, form the backbone of TJs [11] along with cytosolic plaque proteins including zonula occludens (ZO)-1, ZO-2 and ZO-3. The expression profiles of CLDN differ among tissues and determine the tissue-specific paracellular permeability barrier. CLDN6 is localized in cell–cell contacts in the periderm, a protective layer of the developing epidermis, but not in the epidermis [12]. CLDNs 1, 4 and 7 are differentially localized from the basal layer to the granular layer of the epidermis [13] and from the basal to the superficial layer of non-keratinized stratified epithelium [14–20]. TJs are formed in the granular cell layers of the epidermis [21–23] and the superficial cell layer of non-keratinized stratified epithelium, although CLDNs (TJ-forming proteins) are widely localized in the stratified epithelium. In contrast to CLDNs, OCLN is restricted to the granular cell layer of the epidermis [24] or the superficial cell layer of the cornea [14]. Therefore, OCLN, but not CLDNs, is a good marker for TJs.

RA has multiple biological functions in keratinocytes, such as proliferation and differentiation [1]. RA accelerates proliferation in the basal cells and suppresses differentiation, including keratinization (the terminal differentiation of keratinocytes) during migration to the surface of the stratified epithelium. Appropriate amounts of RA are necessary for the maintenance of both keratinized and non-keratinized epithelium *in vivo*; 3D human keratinocyte cultures with RA ranging from 10^–8^ to 10^–9^ M induce the formation of orthokeratinized epithelium, whereas those with less than 10^–9^ M and more than 10^–7^ M RA induced the acceleration and suppression of keratinization, respectively [25]. Retinol deficiency in rats induces the transformation of mucosal non-keratinized epithelium into keratinized epithelium that resembles the epidermis [26]. On the other hand, excessive RA induces a thickened epithelium with widened intercellular space and decreases desmosomes [27–30]. The growth of K38 depends on the serum [31]. Our K38 3D cultures might be under excessive RA conditions because a thickened epithelium with a widened intercellular space was formed. The aim of this study was to investigate whether the inhibition of RAR/RXR heterodimers by HX 531 (an RXR antagonist) in K38 3D cultures affects intercellular junctions, including desmosomes, AJs and TJs without inducing keratinization.

## Materials and methods

### Antibodies

The following primary antibodies were used in this study: mouse anti-OCLN (#33-1500), anti-ZO-1 (#33-9100) and rabbit anti-CLDN1 (#51-9000) antibodies from Zymed (San Francisco, CA, USA); rabbit anti-CLDN4 (ab53156) antibody from Abcam (Cambridge, UK); rabbit anti-CLDN6 (#18865) and anti-CLDN7 (#18875) antibodies from IBL (Takasaki, Japan); rabbit anti-E-cadherin (#3195) antibody from Cell Signaling (San Diego, CA, USA); mouse anti-DSG1 (sc-137164) antibody from Santa Cruz (Dallas, TX, USA); rabbit anti-DSG1 (24587-1-AP) and anti-DSG2 (21880-1-AP) antibodies from Proteintech (Rosemont, IL, USA); mouse anti-DSG3 (D217-3) antibody from MBL (Tokyo, Japan); rabbit anti-DSG3 (LS-C409947) antibody from LifeSpan BioSciences (Seattle, WA, USA); mouse anti-PG (#61005) antibody from Progen (Heidelberg, Germany); rabbit anti-loricrin (LOR) (clone 19 051, #905104) antibody from BioLegend (San Diego, CA, USA) and mouse anti-keratin (K) 4 (WH0003851M1) and rabbit anti-actin (#A2066) antibodies from Sigma-Aldrich (St. Louis, MO, USA).

### Cell culture media

FAD medium (Biochrom GmbH, Berlin, Germany) consisted of Dulbecco’s modified Eagle’s medium (DMEM)/HAM’s F12 (3.5:1.1), 50 μM calcium chloride (CaCl_2_) and 4.5 g/L -glucose and was supplemented with 2.5% Chelex 100-treated fetal bovine serum (FBS), 0.18 mM adenine (Sigma-Aldrich), 0.5 μg/ml hydrocortisone (Sigma-Aldrich), 5 μg/ml insulin (Life Technologies, Carlsbad, CA, USA), 10^–10^ M cholera toxin (Sigma-Aldrich), 10 ng/ml epidermal growth factor (Sigma-Aldrich), 2 mM l-glutamine (Nacalai Tesque, Kyoto, Japan) and 1 mM sodium pyruvate (Wako, Osaka, Japan). Hereafter, this culture is referred to as the ‘complete FAD (c-FAD)’ medium. Serum calcium was removed by treating 500 ml FBS (HyClone, South Logan, UT, USA) with 20 g of Chelex 100 (Bio-Rad Laboratories, Hercules, CA, USA) [32]. For 3D cell cultures [33], the cells were cultured in the c-FAD medium supplemented with 1.2 mM CaCl_2_ (Nacalai Tesque), 10 ng/ml human keratinocyte growth factor (PeproTech, Rocky Hill, NJ, USA) and 0.283 mM l-ascorbic acid phosphate magnesium salt *n*-hydrate (Wako, Osaka, Japan), a stable derivative of ascorbic acid. Hereafter, this is referred to as the ‘FAD-3D medium’.

### 3D cell culture

The murine epidermal keratinocyte cell line, K38 [34,35], originating from the neonatal BALB/c mouse skin, was purchased from the European Collection of Authenticated Cell Cultures (Salisbury, UK). Cells were passaged at a ratio of 1:4 when they reached 70–90% confluency. The culture medium was changed every 2 d. For 3D cell culture, K38 (0.75–1.5 × 10^6^ cells/ml) were seeded in the c-FAD medium into cell culture inserts (0.4 μm polycarbonate filter, 12 mm diameter; Merck Millipore, Darmstadt, Germany) in 24-well plates. Each insert and well contained 0.4 ml cell suspension (3.0–6.0 × 10^5^ cells) and 0.6 ml c-FAD medium. Cells were grown for 1–2 d until they reached 100% confluency. The growth medium inside and outside the insert was replaced with the FAD-3D medium, and cells were cultured for 16–24 h to form intercellular adhesion structures. Then, each insert was transferred to a 12-well plate containing 0.6 ml FAD-3D medium, and airlifted cultures were established by removing the FAD-3D medium in the inserts. The surfaces within the inserts were kept dry following airlift by removing the excess FAD-3D medium in the inserts. The medium was changed every 2 d, and the air-liquid interface culture was maintained for up to 8 d. In some cases, HX 531 or BMS 493, which were from Cayman (Ann Arbor, MI, USA), were added to the FAD-3D medium. Stock solutions of HX 531 and BMS 493 in dimethyl sulfoxide (DMSO) (Nacalai Tesque) were prepared at a concentration of 10 mM.

### Immunofluorescence microscopy

After washing in phosphate-buffered saline (PBS), the 3D cultures were fixed with 1% paraformaldehyde (Wako, Osaka, Japan) in PBS for 1 h at 4°C and washed with PBS. Filters containing cultured cells were removed from the inserts. The filters were sequentially soaked in 10, 20 and 30% sucrose in PBS at 4°C for 1 h each and embedded in an OCT compound (Sakura Finetek Japan, Tokyo, Japan). Cryosections (7 μm) were cut and mounted on glass slides. Some sections were stained with hematoxylin and eosin (HE) (Muto Pure Chemicals, Tokyo, Japan). Cryosections were washed with PBS and incubated with 0.2% Triton-X 100 (Nacalai Tesque) in PBS for 15 min for permeabilization. Subsequently, the sections were washed with PBS and incubated with 1% bovine serum albumin (Sigma-Aldrich) in PBS (BSA–PBS) for 15 min to block any non-specific binding. They were then incubated with primary antibodies diluted in BSA–PBS for 1 h in a moist chamber. The following primary antibodies were used: rabbit anti-CLDN1 (1:100), anti-CLDN4 (1:200), anti-CLDN6 (1:100), anti-CLDN7 (1:100), anti-E-cadherin (1:100), anti-DSG2 (1:100), mouse anti-OCLN (1:50), anti-ZO-1 (1:1000), anti-DSG1 (24587-1-AP) (1:100), anti-DSG3 (D217-3) (1:200), anti-PG (1:50), anti-K4 (1:100) and anti-LOR (1:200) antibodies. After rinsing the sections four times with PBS, they were incubated with an anti-mouse or anti-rabbit immunoglobulin (Ig) conjugated with Alexa 488 or Alexa 568 (Molecular Probes, Eugene, OR, USA) at 1:400 dilution in BSA–PBS for 30 min in the dark. The sections were then washed four times with PBS and mounted in the Vectashield mounting medium containing 4ʹ, 6-diamidino-2-phenylindole (Vector Laboratories, Burlingame, CA, USA). The images were obtained by sequentially scanning the specimen to prevent any bleed-through using an LSM710 confocal laser scanning microscope with ZEN 2010 software (Carl Zeiss, Oberkochen, Germany). Images of the HE-stained sections were acquired using an Olympus BX51 microscope (Olympus Corporation, Tokyo, Japan) with an Olympus objective lens (Ach, 60×/0.80) and an interlaced scan CCD camera (Olympus DP12, 3.24 megapixel, 2048 × 1536 pixels resolution).

### Gel electrophoresis and western blotting analysis

The 3D-cultured cells were washed with ice-cold PBS and lysed with 0.2 ml of lysis buffer [62.5 mM Tris, pH 6.8, 2% sodium dodecyl sulfate (SDS), 10% glycerol, 5% 2-mercaptoethanol and 0.002% bromophenol blue] containing a protease inhibitor cocktail (Sigma-Aldrich) and a phosphatase inhibitor cocktail (Nacalai Tesque). Cell lysates (5 μl per lane) were fractionated by SDS-polyacrylamide gel electrophoresis and transferred to polyvinylidene difluoride membranes. Precision Plus Protein Dual Color Standards (Bio-Rad, Hercules, CA, USA) were used to determine the size of the detected bands. The membranes were incubated with Blocking One (Nacalai Tesque) for 1 h, followed by incubation with primary antibodies overnight at 4°C. Antibodies against CLDN1 (1:1000), CLDN4 (1:1000), CLDN6 (1:1000), CLDN7 (1:1000), E-cadherin (1:2000), ZO-1 (1:1000), DSG1 (sc-137164) (1:2000), DSG2 (1:1000), DSG3 (LS-C409947) (1:1000), PG (1:1000) and actin (1:2000) were used as primary antibodies and diluted with Tris-buffered saline (TBS) [20 mM Tris (pH 7.6) and 137 mM sodium chloride] containing 5% Blocking One buffer. After washing with TBS containing 0.1% Tween 20 (T-TBS), the membranes were incubated with horseradish peroxidase (HRP)-conjugated anti-rabbit or anti-mouse IgGs (1:2000) (GE Healthcare UK Ltd., Amersham, UK) for 1 h. They were then washed with T-TBS and the bands were detected using Luminata Forte Western HRP substrate (Millipore, Billerica, MA, USA). Some membranes were reprobed after stripping the primary and secondary antibodies with the stripping buffer [62.5 mM Tris (pH 6.7), 2% SDS and 100 mM 2-mercaptoethanol] at 50°C for 30 min. Densitometric analysis was performed using the ImageJ 1.53e software (http://rsb.info.nih.gov/ij).

### Measurement of transepithelial electrical resistance

After 8 d of airlift culture, the cell culture inserts were transferred to a 24-well plate. The c-FAD medium containing 1.2 mM calcium was added to the inserts (0.4 ml) and wells (0.6 ml). Transepithelial electrical resistance (TER) was measured using a Millicell ERS-2 Voltohmmeter (Millipore). Subsequently, filters with cultured K38 cells were processed for immunofluorescence microscopy, HE staining, or western blotting analysis. TER values were calculated by subtracting the contribution of the bare filter and medium and multiplying it by the surface area of the filter. All experiments were performed in duplicate.

### Statistical analysis

All data are expressed as the mean ± standard error of the mean. Statistical differences between the groups were determined using the two-sided Welch’s *t*-test. Statistical significance was set at *P* < 0.05.

## Results

### Pharmacological inhibition of RXR improved the morphology of K38 3D cultures but did not induce orthokeratinization

**Fig. 1.**
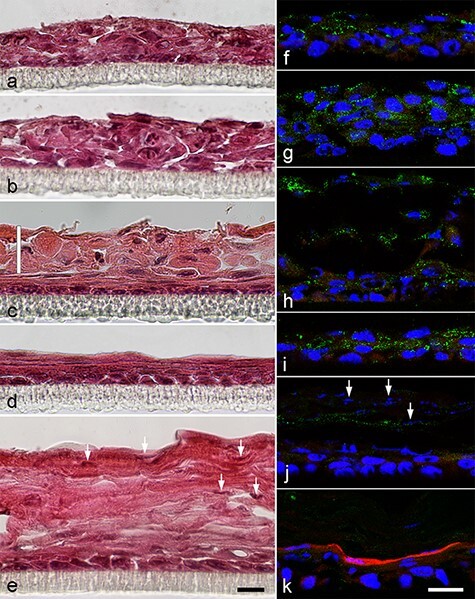
Examination of morphology and differentiation in K38 3D cultures treated with HX 531 or BMS 493. K38 seeded on the filters of cell culture inserts were airlifted for 8 d with 0 (a, f), 0.1 (b, g), 0.2 (c, h), 0.5 (d, i) or 1.0 μM HX 531 (e, j) or with 0.2 μM BMS 493 (k). Morphology of 3D cultures was examined after hematoxylin-eosin (HE) staining (a–e). Localization of differentiation markers, K4 (green) and LOR (red) was examined by double immunofluorescence staining (f–k). The reconstituted epithelium was non-keratinized and thick, and the superficial cells were swollen rather than squamous in 0 (a) and 0.1 μM HX 531-treated cultures (b). When treated with 0.2 μM HX 531 (c), superficial cells were found to be flattened in the lower half of the structures of the stratified squamous epithelium. In contrast, cells in the upper half of the structures (vertical white line in c) consisted of swollen cells with large gaps between cells. Treatment with 0.5 μM HX 531 (d) formed non-keratinized stratified squamous epithelium, whose superficial cells were flattened. Treatment with 1.0 μM HX 531 (e) formed the cornified layer-like structures with considerable number of nuclei (arrows). Large gaps were frequently observed in the epithelium (a, b) or in the upper half of the epithelium (c). These large gaps were hardly observed when treated with 0.5 (d) or 1.0 μM (e) HX 531. K4 was detected in suprabasal cells, but LOR was not detected in 0, 0.1, 0.2 or 0.5 μM HX 531-treated cultures (f–h). When treated with 1.0 μM HX 531, weak staining for K4 and 4ʹ, 6-diamidino-2-phenylindole staining for nuclei (arrows) were observed in the cornified layer-like structures, but LOR was not detected (j). When treated with 0.2 μM BMS 493 (k), LOR was detected in cells just below the cornified layer, but K4 was not detected. Black scale bar: 20 μm for a–e, White scale bar: 20 μm for f–k.

K38 3D cultures reconstituted non-keratinized, stratified epithelium-like structures. However, the superficial cells were swollen rather than squamous in the presence of 0, 0.1, or 0.2 μM HX 531 (Fig. 1a–c). When treated with 0.2 μM HX 531 (Fig. 1c), the lower half of the structures resembled stratified squamous epithelium *in vivo*, while the upper half of the structures (vertical white line in Fig. 1c) consisted of swollen cells with large gaps between cells, as seen when treated with 0 (Fig. 1a) or 0.1 μM HX 531 (Fig. 1b). Treatment with 0.5 μM HX 531 (Fig. 1d) formed non-keratinized stratified squamous epithelium without large gaps between cells, which resemble *in vivo* epithelium. Treatment with 1.0 μM HX 531 (Fig. 1e) formed cornified layer-like structures with some nuclei (arrows in Fig. 1e) on the surface of the epithelium. Differentiation of keratinocytes was examined by double immunostaining for K4 and LOR (Fig. 1f–k). Keratin 4, a marker for suprabasal cells in non-keratinized stratified epithelium, was detected in suprabasal cells when treated with 0, 0.1, 0.2, or 0.5 μM HX 531 (Fig. 1f–i). When treated with 1.0 μM HX 531 (Fig. 1j), a considerable number of fragmented nuclei and weak K4-positive signals, but not LOR-positive signals (a marker for keratinized epithelium detected in the granular layer), were detected in the thick surface layer, suggesting that the reconstituted structures were not orthokeratinized epithelium. When treated with 0.2 μM BMS 493 (a pan-RAR inverse agonist), LOR was detected but K4 disappeared, suggesting that the reconstituted structures were orthokeratinized epithelium. Inhibition of RAR/RXR by HX 531 improved the morphology of the reconstituted epithelium in a dose-dependent manner; it tightened intercellular spaces and decreased thickness of the epithelium, although treatment with high doses of HX 531 (1.0 μM) did not induce orthokeratinization (terminal differentiation of keratinocytes).

### Inhibition of RXR increased the levels of desmosomal and TJ proteins in K38 3D cultures

**Fig. 2.**
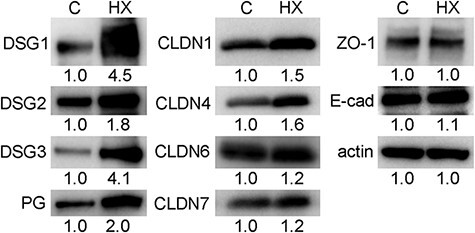
Inhibition of RXR increased the expression levels of the desmosomal and TJ proteins. After densitometric analysis of western blotting, protein levels were normalized to the corresponding actin levels and showed relative to the amount present in the control cultures (C) below the blots. Protein levels of desmoglein (DSG)-1, DSG2, DSG3, plakoglobin (PG), claudin (CLDN)-1, CLDN4, CLDN6 and CLDN7 in HX 531-treated cultures (HX) were 4.5, 1.8, 4.1, 2.0, 1.5, 1.6, 1.2 and 1.2 times higher than those in control cultures, respectively. Protein levels of zonula occludens-1 (ZO-1) and E-cadherin were not changed by HX 531 treatment.

We examined the protein expression of TJs, AJs and DSMs by western blotting (Fig. 2) because many gaps were observed except for treatment with 0.5 μM HX 531. When treated with 0.5 μM HX 531, protein expression of DSMs, DSG1, DSG2, DSG3 and PG, increased to 4.5, 1.8, 4.1 and 2.0 times higher compared in the control, respectively. Protein expression of TJs, CLDN1, CLDN4, CLDN6 and CLDN7 increased to 1.5, 1.6, 1.2 and 1.2 times higher compared in the control, respectively, while the protein expression of ZO-1 was not altered. Protein expression of AJs, E-cadherin, was slightly increased to 1.1 times higher compared in the control.

### Inhibition of RXR restored the layer-specific localization of desmosomal proteins in K38 3D cultures

**Fig. 3.**
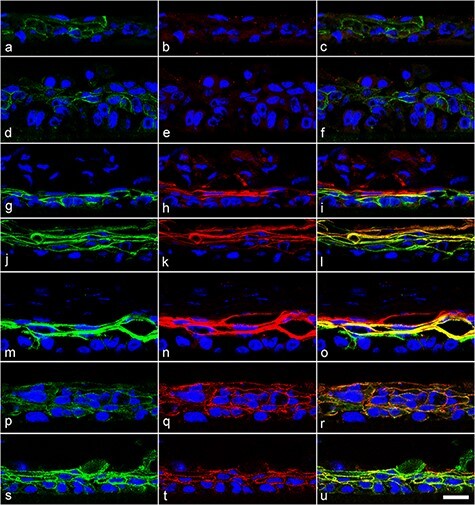
Inhibition of RXR restored the layer-specific localization of DSG1, DSG3 and PG. K38 cultures were airlifted for 8 d with 0 (a–c, p–r), 0.1 (d–f), 0.2 (g–i), 0.5 (j–l, s–u) or 1.0 μM HX 531 (m–o) and double-immunostained with DSG3 (green) and DSG1 (red) (a–o) or with PG (green) and E-cadherin (red) (p–u). Merged images are shown in c, f, i, l, o, r and u. Nuclei were stained with 4ʹ, 6-diamidino-2-phenylindole (blue). Weak signals for DSG3 (a) and PG (p), and no signals for DSG1 (b) were detected in the cell–cell contacts of suprabasal cells in the absence of HX 531. Similarly, weak signals for DSG3 (d) and no signals for DSG1 (e) were detected when treated with 0.1 μM HX 531. When treated with 0.2 μM HX 531 (g–i), intense signals for DSG1 and DSG3 were observed in the lower half of the reconstituted epithelium, but only weak signals for DSG1 were observed in the upper half of the epithelium. When treated with 0.2 (g–i), 0.5 (j–i) and 1.0 μM (m–o) HX 531, both DSG1 and DSG3 was detected in cell–cell contacts of suprabasal cells, but DSG1 was localized in the upper layers of suprabasal cells compared to DSG3. Intense signals for PG in cell–cell contacts were detected in suprabasal cells when treated with 0.5 μM HX 531 (s–u). E-cadherin was consistently localized from the basal layer to the superficial layer with or without HX531 (q, t). Scale bar: 20 μm for all images.

When primary antibodies were omitted, no specific signals were observed with or without HX 531 (Supplementary Fig. S1a–c and g–i). We examined the localization of DSG2, but we could not detect any specific signals in cell–cell contacts with or without HX 531 (Supplementary Fig. S1e and k). ZO-1 was localized in the superficial and suprabasal cells with or without HX 531 (Supplementary Fig. S1d and j). Next, we examined the localization of the DSM-constitutive proteins DSG1, DSG3 and PG (Fig. 3). Weak signals for DSG3 (Fig. 3a) and PG (Fig. 3p) and no signals for DSG1 (Fig. 3b) were detected in cell–cell contacts of suprabasal cells in the absence of HX 531. Similarly, weak signals for DSG3 (Fig. 3d) and no signals for DSG1 (Fig. 3e) were detected when treated with 0.1 μM HX 531. When treated with 0.2 μM HX 531 (Fig. 3g–i), intense signals for DSG1 and DSG3 were observed in the lower half of the reconstituted epithelium and weak signals for DSG1 were observed in the upper half of the epithelium. When treated with 0.2 (Fig. 3g–i), 0.5 (Fig. 3j–i) and 1.0 μM (Fig. 3m–o) HX 531, both DSG1 and DSG3 were detected in the cell–cell contacts of suprabasal cells, but DSG1 was localized in the upper layers of suprabasal cells compared to DSG3. Intense signals for PG in cell–cell contacts were detected in suprabasal cells when treated with 0.5 μM HX 531 (Fig. 3s–u). E-cadherin was consistently localized from the basal layer to the superficial layer, with or without HX531 (Fig. 3q and t).

### Inhibition of RXR recruited CLDN1 to OCLN-positive cell–cell contacts in the superficial cells

**Fig. 4.**
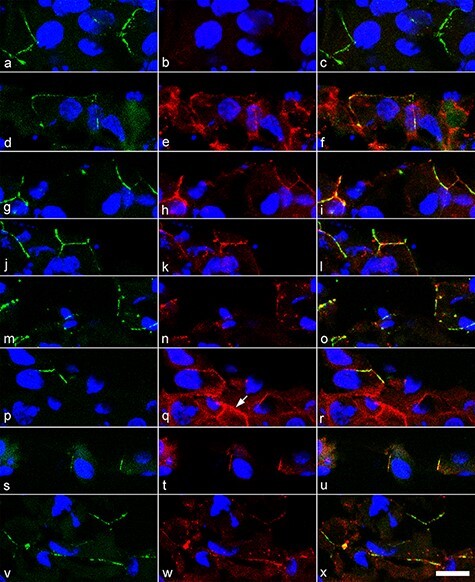
Inhibition of RXR recruited CLDN1- to OCLN-positive cell–cell contacts in the superficial cells. K38 cultures were airlifted for 8 d without HX 531 (a–l) or with 0.5 μM HX 531 (m–x). Oblique sections were double-immunostained with OCLN (a, d, g, j, m, p, s and v) and either CLDN1 (b, n), CLDN4 (e, q), CLDN6 (h, t) or CLDN7 (k, w). Merged images are shown in c, f, i, l, o, r, u and x. Nuclei were stained with 4ʹ, 6-diamidino-2-phenylindole (blue). CLDN1 was not detected in OCLN-positive cell–cell contacts in the superficial cells in the control (b), but it was detected in the HX 531-treated cultures (n). OCLN-positive cell–cell contacts in the superficial cells were colocalized with CDN4 (e, q), CLDN6 (h, t) and CLDN7 (k, w) in the absence or presence of HX 531. CLDN4 was detected in cell–cell contacts below the superficial cells where OCLN was not colocalized (arrow) when treated with HX531 (q). Scale bar: 20 μm for all images.

The localization of TJ proteins, including OCLN, CLDN1, CLDN4, CLDN6 and CLDN7 was examined (Fig. 4). OCLN was detected in cell–cell contacts of the superficial cells (Fig. 4a, d, g, j, m, p, s and v). CLDN1 was not detected in OCLN-positive cell–cell contacts in the control (Fig. 4b) but was detected in the presence of HX 531 (Fig. 4n). CLDN4 (Fig. 4e and q), CLDN6 (Fig. 4h and t) and CLDN7 (Fig. 4k and w) were colocalized with OCLN-positive cell–cell contacts in the absence or presence of HX 531. CLDN4 was also observed in cell–cell contacts of suprabasal cells in the presence of HX531 (arrow in Fig. 4q).

### Inhibition of RXR increased the TER

**Fig. 5.**
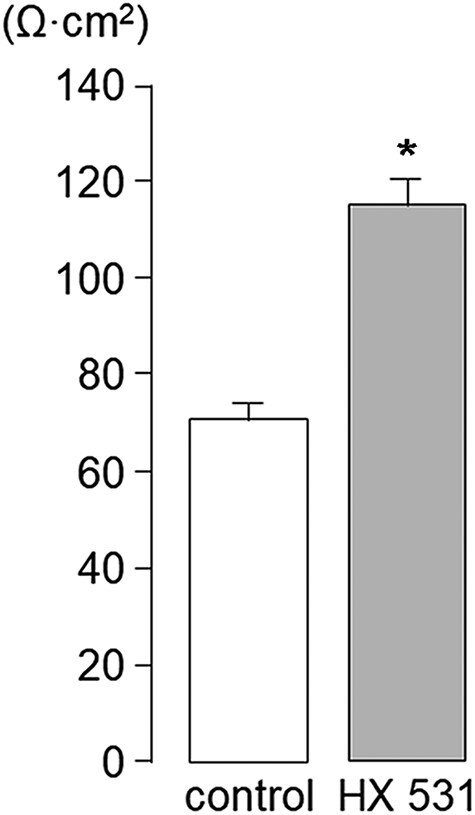
Inhibition of RXR increased the TER in K38 3D cultures. K38 cultures were airlifted for 8 d without HX 531 (control) or with 0.5 μM HX 531, and the TER was measured. TER values in the control and HX 531-treated cultures were 71.1 ± 3.0 Ω· cm^2^ and 115.5 ± 5.1 Ω· cm^2^, respectively. HX 531 treatment resulted in a 62.4% increase in TER compared with the control. Values are expressed as the mean ± standard error of the mean. *P < 0.01 vs control.

HX 531 treatment improved the morphology of 3D cultures, increased protein expression of DSMs and TJs, restored localization of desmosomal proteins resembling a non-keratinized stratified epithelium *in vivo* and recruited CLDN1 to TJs. Based on these results, we speculated that the paracellular permeability barrier of 3D cultures might increase by HX 531 treatment. HX 531 treatment resulted in a 62.4% increase in TER compared to the control (Fig. 5).

## Discussion

K38 3D cultures in serum-containing medium formed a non-keratinized stratified epithelium, whose morphology was not similar to that *in vivo*. The formed epithelium was thick with swollen cells in the surface layer and showed a loosened appearance of cell–cell contacts, probably due to the disrupted layer-specific localization of DSG1, DSG3 and PG. Our observations are consistent with those of previous reports. RA influences differentiation, as revealed by the suppression of the expression of differentiation markers, including keratin K1, K10, loricrin, filaggrin and transglutaminase, which show layer-specific distribution in stratified epithelium [36]. RA also inhibited keratinization [27,28,37], which occurs in the upper layers of the epidermis by forming a cornified envelope [38], and helped the keratinocytes become flattened in the uppermost cells. Therefore, RA derived from serum induced thick non-keratinized epithelium with some large gaps in control K38 3D cultures.

It is currently unknown why 0.2 μM BMS 493 induced orthokeratinization but 1.0 μM HX 531 did not. Heterodimers of RXR and another nuclear receptor cause non-permissive, permissive, or conditional permissive transactivation [39–41]. Conditional permissive heterodimers, such as RAR/RXR, preclude the binding of the RXR ligand. Binding of the ligand to RAR causes RAR/RXR activation and allows binding of the RXR ligand to RXR, and RAR/RXR functions in a synergistic manner. RAR/RXR can be activated solely by binding of the RAR ligand to RAR, but its transcriptional response is weak. We speculated that a weak transcriptional response, which suppresses orthokeratinization, may occur before inhibition of RAR ligand-bound RAR/RXR by HX 531.

HX531 may also inhibit heterodimers such as LXR/RXR, PPAR/RXR, VDR/RXR and TR/RXR in keratinocytes and may affect keratinocyte differentiation and permeability barrier. In fact, HX531 inhibits the troglitazone (a PPARγ agonist)-induced K13 expression [42]. It is suggested that LXR ligands could induce activator protein 1 (a transcription factor)-dependent expression of keratinocyte differentiation genes [43]. PPAR and LXR activation increases expression of ATP-binding cassette (ABC) transporters such as ABCA1 and ABCA12, which is involved in lipid transport, and improves permeability barrier by stimulating keratinocyte differentiation and lipid transport [44,45]. Therefore, the possibility of inhibition of RXR/nuclear receptors (RAR, LXR, PPAR, VDR and TR) by HX 531 may cause different results between the treatment of HX 531 and BMS 493. The detailed mechanism of action of HX 531 on K38 is currently unknown, and further research is required.

When treated with 0.2 μM HX 531, double layers were formed; the lower layer looked like normal non-keratinized epithelium, and the upper layer consisted of large swollen cells with some large gaps between cells (Fig. 1c). A thick epithelium was formed, probably due to RA-induced hyperproliferation. Treatment of 0.2 μM, but not 0.1 μM, HX 531 may suppress hyperproliferation and normalize the epithelial morphology, but swollen cells in the upper layer, which were destined to exfoliate, remained on the epithelial surface. The swollen cells in the upper layer may be attached to the lower layer and to each other by some cell–cell adhesion molecules, including DSG1, which was detected in the upper layer (Fig. 3h).

Cell–cell contacts in control cultures showed a loosened appearance with swollen cells in the surface layer compared with those in RXR-inhibited cultures. These results suggested that RAR/RXR-mediated signaling affected the formation of cell–cell contacts and flattening of the superficial cells. Western blotting showed that RXR inhibition increased the expression of desmosomal proteins, such as DSG1, DSG2, DSG3 and PG, but did not change the expression of the AJ protein, E-cadherin. In addition, neither DSG1, DSG3, nor PG was localized in some cell–cell contacts, even though E-cadherin was localized. Based on these results, RAR/RXR-mediated signaling inhibited the formation of DSMs, which is indicated by the concentration of DSG1, DSG3 and PG in cell–cell contacts but not the formation of AJ. Consistent with our results, RA loosened cell–cell contacts by decreasing the number of DSMs in mouse skin *in vivo* [27], organotypic human skin cultures [28], HaCaT keratinocytes [29] and GE1 cells (mouse gingival keratinocytes) [30]. Protein and mRNA expression of DSM-related molecules, including DSG1, DSG3, desmocollin (DSC) 1, DSC2 and DSC3, was decreased by RA treatment [29,30]. Taken together, loosening of cell–cell contacts induced by RAR/RXR-mediated signaling is probably attributed to a decrease in desmosomal proteins but not E-cadherin.

In this study, we found that RXR inhibition restored the layer-specific localization of DSG1, DSG3 and PG in K38 3D cultures; they were localized in the upper layers of suprabasal cells (DSG1) or the whole suprabasal cells (DSG3 and PG). It is reasonable that the distribution of PG overlapped with that of DSG1 and DSG3 because PG binds to DSGs [8]. In control cultures, they were heterogeneously distributed in suprabasal cells with a dotted appearance rather than a linear appearance. Consistent with the results of western blotting, the staining intensity in control cultures was weaker than that in RXR-inhibited cultures. In previous reports [9,10], DSG1 was detected throughout the epithelium (both epidermis and mucosal epithelium), much more intense in the superficial layers, whereas DSG3 was restricted to the basal and most immediate suprabasal cells in the epidermis or localized throughout the mucosal epithelium. Taken together, RXR inhibition normalized the distribution of DSG1, DSG3 and PG, resembling the mucosal epithelium rather than the epidermis.

Freeze-fracture electron microscopy demonstrated that TJs are recognized as intramembranous particles (TJ strands) or complementary grooves in the protoplasmic or exoplasmic face, respectively. OCLN alone could not form TJ strands when exogenously expressed in TJ-free fibroblasts, whereas CLDN1 alone could form TJ strands [11]. OCLN was co-polymerized into TJ strands formed by CLDN1 when both OCLN and CLDN1 were expressed in fibroblasts [11]. Therefore, OCLN can be used as a marker of TJ formation.

CLDN1 was not detected in OCLN-positive cell–cell contacts in non-keratinized epithelium reconstituted by K38 3D cultures, while RXR inhibition restored CLDN1 in OCLN-positive cell–cell contacts. In the reverse case, CLDN1 disappeared from OCLN-positive cell–cell contacts when COCA 3D cultures were treated with atRA, which changed the keratinized epithelium to non-keratinized epithelium [37]. RA also decreased the mRNA and protein expression levels of CLDN1 in human epidermal keratinocytes, mouse epidermis and mouse gingival keratinocytes [46,47]. It is believed that CLDNs form pores or channels with a sealing function to reduce permeability (such as CLDN1) or to enhance permeability [48–50]. The functions of CLDN4 and CLDN7 are inconsistent, and the function of CLDN6 is unknown [48–50]. Overexpression of CLDN1 in MDCK II cells increased TER [51]. Claudin-1-deficient mice, whose TJs in the epidermis were localized with OCLN and CLDN4, died at birth due to water loss from the epidermis [21]. Inhibition of RXR increased the protein expression of CLDN1 and CLDN4 in this study. However, increased CLDN4 was localized in cell–cell contacts in suprabasal cells without colocalization with OCLN. Therefore, increased CLDN1 levels in TJs may contribute to increased TER, although the contribution of CLDN4, CLDN6 and CLDN7 to permeability remains undetermined.

## Concluding remarks

We improved K38 3D cultures by inhibiting RAR/RXR-mediated signaling using 0.5 μM HX 531, which maintained non-keratinization. RXR inhibition induced the narrowing of intercellular spaces accompanied by improved morphology, restoration of layer-specific distribution of DSG1 and DSG3 and decrease in paracellular permeability accompanied by recruitment of CLDN1 to OCLN-positive cell–cell contacts, but did not influence the protein expression and localization of E-cadherin. Therefore, K38 3D cultures treated with 0.5 μM HX531 provides a useful in vitro model to study intercellular junctions in the non-keratinized epithelium.

## Supplementary Material

dfac007_Supp
