# Supplementary figures and images for: Inhibition of retinoid X receptor improved the morphology, localization of desmosomal proteins and paracellular permeability in three-dimensional cultures of mouse keratinocytes
Source: Microscopy (Oxf). 2022 Feb 16;71(3):152–60. doi: 10.1093/jmicro/dfac007 (PMC9169536; doi:10.1093/jmicro/dfac007)

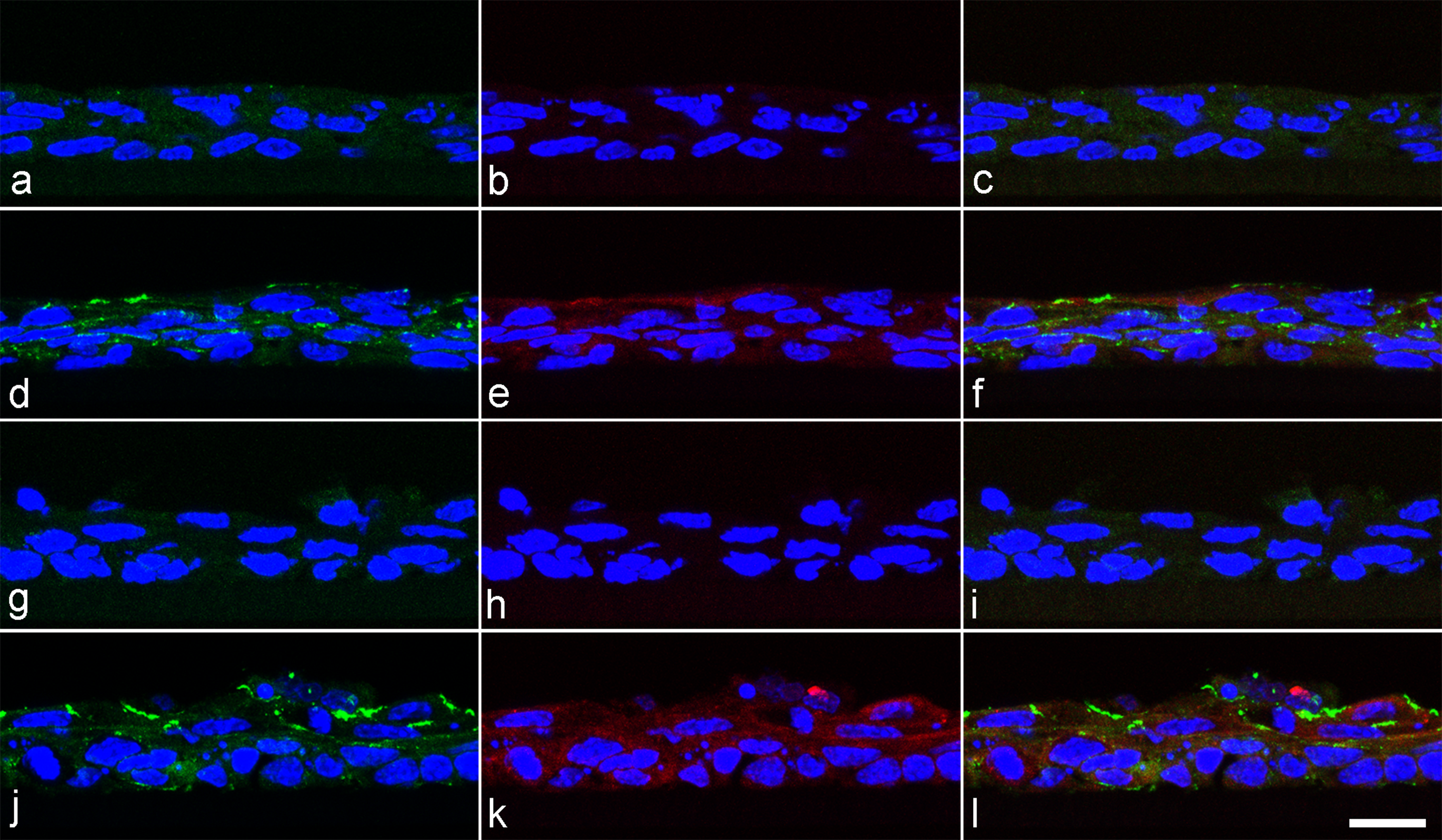

Supplement: dfac007_Supp [file dfac007_supp.zip › new Suppl Fig1-1.tif]
